# Psychometric analysis of the social connectedness instrument

**DOI:** 10.3389/fpsyg.2025.1565267

**Published:** 2025-07-11

**Authors:** Brian Kelley, Blake Fraser, Anessa Wells, Matthew Ferdock

**Affiliations:** The Social Connectedness Lab, Department of Psychology, Liberty University, Lynchburg, VA, United States

**Keywords:** loneliness, social connectedness, social disconnectedness, psychometric validation, structural equation modeling, latent constructs, intervention, young adults

## Abstract

**Introduction:**

Social connectedness is decreasing, especially among young adults, which poses a significant public mental and physical health threat globally. However, before attempting to improve social connectedness, measurement must first be evaluated. Many previous instruments used to measure loneliness (perceived social disconnectedness) provide a simple measure of intensity (i.e., how lonely/disconnected someone feels) but lack information about specific factors of loneliness and disconnectedness that are potentially modifiable (e.g., social skills, negative thoughts, technology use).

**Methods:**

The current study aims to address these gaps by evaluating the psychometric properties of the Social Connectedness Instrument (SCI) using a sample of 719 college students (Mage = 19.63, SDage = 1.60, 75% female) attending a mid-Atlantic university. Psychometric evaluation, including exploratory factor analysis, principal component analysis, structural equation modeling, and tests of reliability and validity, was performed on the SCI.

**Results:**

The final model of the SCI consists of two latent constructs, Psychoemotional Disconnectedness (PED) and Psychosocial Disconnectedness (PSD), which displayed satisfactory psychometric properties overall. While PED refers to a perception of feeling disconnected due to emotional contributors (e.g., social anxiety, fear of rejection), PSD refers to a perception of feeling disconnected due to social contributors (e.g., social skills, social motivation). An additional eleven single-item indicators of disconnectedness, which were not included in the final model, may be retained for further insight into someone's disconnectedness if brevity is not an issue.

**Discussion:**

This novel instrument is recommended for use when a greater depth of perceived social disconnectedness and potentially modifiable contributors are needed to inform individualized interventions.

## 1 Introduction

Social connection/connectedness is a multidimensional construct that is defined as a continuum of the size and diversity of one's social network and roles, the functions that these relationships serve, and their positive or negative qualities (Holt-Lunstad, 2024, 2025). If one of these aspects of social connection are not achieved, a person may experience loneliness, a lack of social support, or a diminished social network. A recent scientific and public health emphasis has been placed on loneliness (perceived social isolation/disconnectedness), which is a particular dimension of social connectedness. While social connectedness is often defined objectively (e.g., social network size, social isolation; Holt-Lunstad, 2024), loneliness is defined as the perceived discrepancy between a person's desired and actual quantity and/or quality of relationships, resulting in a negative emotional state (Perlman and Peplau, 1981). Acute feelings of loneliness are universal and adaptive, leading individuals to reconnect with others (Cacioppo et al., 2010; Ypsilanti and Lazuras, 2022). In contrast to this, chronic feelings of loneliness are harmful and maladaptive, resulting in further perceived isolation, worse social connection, and negative affect (Cacioppo et al., 2010; Ypsilanti and Lazuras, 2022). Due to the global prevalence of these issues (Surkalim et al., 2022), loneliness and a lack of adequate social connection are being considered a public health threat. In the U.S., the Surgeon General declared that there is a “loneliness epidemic,” citing that changes must occur at a systemic level to improve social connection and reduce the adverse effects of loneliness (Murthy, 2023). However, until loneliness and poor social connectedness are better understood and conceptualized, it will be difficult to prevent and mitigate the growing problem.

Studies have reported that the prevalence of loneliness when accounting for age appears to be a U-shaped distribution, which indicates that loneliness is highest among late adolescents/young adults and older adults (Lasgaard et al., 2016; Victor and Yang, 2012). A recent meta-analysis on longitudinal studies reported this relationship between age and loneliness over time (Graham et al., 2024); participants had higher loneliness during late adolescence/young adulthood, followed by lower levels of loneliness during middle adulthood and steep increases in loneliness during older adulthood (Graham et al., 2024). Furthermore, other studies have shown that participants under 25 reported the highest rate (9%) of severe loneliness (Victor and Yang, 2012), and there was a 50% increase in agreeing to often feeling lonely for 12th graders in 2017 compared to 12th graders in 2012 (Twenge et al., 2019). Studies focused on social disconnectedness and social support have reported similar trends. Bruss et al. (2024) found that 24.1% of adults on average reported rarely or never receiving the social and emotional support they needed, with the highest rate (29.7%) reported by young adults (18–34). Among adolescents, 38.5% expressed feeling disconnected from others at school (Wilkins et al., 2023), with social ties appearing to decline with age (Akindele and Adebayo, 2021). Thus, while loneliness and poor social connectedness are important issues to address for all ages, they are an increasingly important issue to address within the late adolescent/young adult population. If chronic feelings of loneliness and poor social connectedness are left unaddressed, individuals are vulnerable to physical and mental health problems across the lifespan (Hawkley and Cacioppo, 2010).

Deficits in social connectedness can have significant adverse biopsychosocial consequences across the lifespan. Previous research has shown relationships between poor social connectedness (high loneliness, high social isolation, poor relationship quality) and various physical health problems, such as increased risk for hypertension, heart disease, stroke, and premature mortality (Holt-Lunstad et al., 2015, 2017; Park et al., 2020; Valtorta et al., 2016; Ueno et al., 2022). These relationships may exist due to an impact on key biological processes. For example, high levels of loneliness are related to higher levels of cortisol, inflammation, dysregulated immunity, a dysregulated hypothalamic-pituitary-adrenal axis, and worse health behaviors (Cacioppo et al., 2011; Cole et al., 2007; Lauder et al., 2006). Additionally, high loneliness and low perceived social support have strong relationships with worse mental health and lower emotional wellbeing (Park et al., 2020; Wickramaratne et al., 2022). Loneliness is linked with (cross-sectionally and longitudinally) higher levels of depression, anxiety, social anxiety, and suicidal ideation (Allan et al., 2021; Cacioppo et al., 2010; Helm et al., 2020; Maes et al., 2019; Moeller and Seehuus, 2019; Park et al., 2020; Shaw et al., 2021). Loneliness is also related to poorer social functioning, such as inhibiting a person's positive affect, enjoyment, and responsivity they demonstrate during a social interaction (Arpin and Mohr, 2018; Moeller and Seehuus, 2019; Smith et al., 2022).

The culmination of recent research findings indicate that poor social connectedness is a growing public health threat, negatively impacting overall biopsychosocial quality of life and wellbeing. Thus, we propose that social connectedness should be assessed as an essential marker of an individual's wellbeing by physicians, counselors, psychologists, teachers, employers, religious leaders, and community organizers. However, measuring an individual's level/intensity of social connectedness is only the starting point of what needs to be addressed. Subsequent efforts need to be made to reduce chronic feelings of disconnectedness and promote increased social connection. Previous research on efforts (i.e., interventions) to reduce chronic feelings of loneliness have shown mixed results (small to moderate effects) depending on type of intervention (e.g., social skills, social support, social contact, psychological, school-based) (Eccles and Qualter, 2021; Hickin et al., 2021; Masi et al., 2011; Allen et al., 2021). Due to the complexities of perceived social disconnectedness and varying perceptions on what might be contributing to or causing the experience (Hawkley and Cacioppo, 2010; Hemberg et al., 2022; Turner et al., 2024), researchers suggest providing an intervention plan tailored to an individual's unique experience with the phenomenon (Eccles and Qualter, 2021; Hickin et al., 2021; Masi et al., 2011). For example, if someone is experiencing disconnectedness due to poor interpersonal social skills, a social skills intervention may be the most appropriate for reducing feelings of loneliness and promoting social connection. If someone is experiencing disconnectedness due to maladaptive social cognitive processes (e.g., negative thoughts about themselves or others, social anxiety), then a psychological intervention may be more appropriate. However, to our knowledge, no social connectedness instrument exists to provide insight into someone's intensity of perceived disconnectedness (i.e., loneliness) in addition to potentially modifiable contributing variables (e.g., social skills, social anxiety) to that experience.

Well-established social connectedness instruments include, but are not limited to, the following: the Loneliness and Aloneness Scale for Children and Adolescents (LACA; Marcoen et al., 1987); the Children's Loneliness and Social Dissatisfaction Scale (CLS; Asher et al., 1984); the UCLA Loneliness Scale (UCLA LS; Russell et al., 1978; Russell, 1996), the Differential Loneliness Scale (DLS; Schmidt and Sermat, 1983), the Social and Emotional Loneliness Scale for Adults (SELSA; DiTommaso and Spinner, 1993), the De Jong Gierveld Loneliness Scale (DJGLS; de Jong-Gierveld and Kamphuis, 1985), the Revised Social Connectedness Scale (SCS-R; Lee et al., 2001), the Watts Connectedness Scale (WCS; Watts et al., 2022), the Hemmingway Measure of Adolescent Connectedness (HMAC; McWhirter and McWhirter, 2011), the Multidimensional Scale of Perceived Social Support (MSPSS; Zimet et al., 1988), the Sense of Belonging Instrument (SOBI; Hagerty and Patusky, 1995), and the Basic Psychological Needs Satisfaction Scale (BPNS; Ryan and Deci, 2000). The UCLA LS is the most widely used instrument in the scientific literature (Cole et al., 2021; Maes et al., 2022), and it attempts to provide a unidimensional measure for feelings of loneliness. Other instruments, such as the LACA (Marcoen et al., 1987), SELSA (DiTommaso and Spinner, 1993), DJGLS (de Jong-Gierveld and Kamphuis, 1985), SCS-R (Lee et al., 2001), WCS (Watts et al., 2022), HMAC (McWhirter and McWhirter, 2011), and MSPSS (Zimet et al., 1988) attempt to provide a multidimensional measure for various latent constructs of connectedness (e.g., social loneliness, emotional loneliness, perceived familial support, perceived peer support, etc.) (Too et al., 2022). More recently, researchers have used brief measures to quickly capture the intensity of perceived social disconnectedness (i.e., loneliness), especially in population-based studies (de Jong-Gierveld and Tilburg, 2006; Halvorson and Kuczynski, 2024; Hughes et al., 2004; Kotwal et al., 2022). Overall, these measures provide valuable insight into the intensity of social connectedness and various latent constructs, but there is limited insight provided about potential contributors to one's experience of disconnectedness. Additionally, while all of these measures focus solely on perceived social disconnectedness (i.e., loneliness, perceived belonging, perceived social support), social connection also includes objective aspects (Holt-Lunstad, 2025). Thus, there is need for a novel social connectedness instrument that provides more information about an individual's personalized experience of perceived disconnection and isolation in addition to objective modifiable contributors (e.g., number of close relationships, meaningful conversations, technology use) that could help in future intervention implementation.

The purpose of the current study is to assess the psychometric properties of a novel instrument, the Social Connectedness Instrument (SCI), designed to assess not only intensity of perceived social disconnectedness but also important perceived contributors (both subjective and quantifiable/objective) to that experience. For example, on the UCLA LS (version 3; Russell, 1996) a sample item is, “How often do you feel alone?” which provides insight into intensity of loneliness but limited knowledge from a modification standpoint (i.e., how to deal with the loneliness/disconnectedness). However, the SCI consists of subjective items, like “I am concerned people will reject me” and “Because of the heavy emotions I experience, I withdraw from others,” and objective/quantifiable items, like “How many close relationships do you have?” and “How many hours a day do you normally spend watching television, movies, or streaming content?” which provide insight into potentially modifiable contributors (e.g., fear or rejection, heavy emotions, close relationships, watching/streaming entertainment) to difficulties in connecting with others. While other perceived social disconnectedness instruments still have immense value for capturing feelings of loneliness, social support, and belongingness, especially in population-based research, the SCI may provide more details and insight into an individual's specific experience with feelings of loneliness and disconnection.

## 2 Aims

Our study aimed to assess the reliability and validity of the SCI in a sample of young adults at a university. Our objectives were as follows:

1. Conduct exploratory factor analysis and structural equation modeling to determine the latent constructs of the instrument.
2. Assess the fit and internal reliability and validity of the proposed model.
3. Examine the external convergent and divergent validity of the proposed model.

## 3 Materials and methods

### 3.1 Item development and data collection

The first iteration of items for the SCI was developed using an inductive and deductive strategy (Swan et al., 2023). We conducted focus-groups and qualitative interviews to establish perceptions of social connectedness (loneliness, social support, social isolation) and its causes and contributors amongst the target audience (young adults). Subsequently, we performed a literature review to cross-validate these perceptions. For example, in the interviews, many individuals related feelings of poor social connectedness to fear of rejection, which we then confirmed in the literature. Initially, 23 items were developed for the SCI, and pilot data was collected from 367 university students on these items. After assessing the initial data and receiving feedback on the content validity of these items, the following changes were made: (1) several items were edited for clarity, (2) several items were deleted due to poor content validity, and (3) several items were added to increase content validity.

The final iteration of the SCI contained 26 items (Table 1). Items 1–18 were rated on a Likert-type scale from one (never) to seven (always) and contained items on perceived social disconnectedness and potential contributors to the experience. Items 19–25 were rated on a quantitative scale from zero to five (or more) and contained objectively quantifiable aspects of social connection. Item 26 was rated on a Likert-type scale from one (not at all lonely) to ten (extremely lonely) and provided a single-item measure of acute feelings of loneliness. The data collection for the current study consists of three different waves. All three waves of data collection included the SCI but contained different secondary measures. The first wave consisted of 348 university students measured on the SCI and self-reported health behaviors. The second wave consisted of 91 university students measured on the SCI, social anxiety, and wellbeing (*N* = 43 completed a second time point after 7 days). The third wave consisted of 292 university students measured on the SCI and mindfulness. All of the data across studies were collected via Qualtrics and subsequently combined in Microsoft Excel to create the total sample for the current study.

**Table 1:** The Social Connectedness Instrument.

**Item #**	**Statement**	**Category**
**Description: The Social Connectedness Instrument assesses a person's perceived social connectedness and behavioral components of connection. In total, the instrument contains 26 items and questions split up into three sections.**		
**Instructions: Select the response that most accurately describes you or your situation. The scale consists of 1–7: 1** = **Never**, **2** = **Very Rarely, 3** = **Rarely, 4** = **Sometimes, 5** = **Often, 6** = **Very Often, 7** = **Always**		
1	Where I live limits my ability to have consistent positive social interactions.	Single-Item
2	Recent transitions have made me feel more socially connected. **(Reverse scored)**	PSD
3	Health concerns make it difficult for me to connect with others.	PED
4	I am concerned people will reject me.	PED
5	My interests, desires, or hobbies are shared by the people around me. **(Reverse scored)**	PSD
6	I feel confident in my ability to initiate relationships with others. **(Reverse Scored)**	PSD
7	I feel anxious in social situations.	PED
8	The risk of being bullied outweighs my desire to build friendships.	PED
9	My commitments (e.g., work, school, extracurriculars) interfere with my social life.	Single-Item
10	I think I am treated poorly by my friends.	PED
11	I lack the motivation to invest in deeper relationships.	PSD
12	To fit in, I change how I act around people.	PED
13	I feel included by others. **(Reverse scored)**	PSD
14	I prefer connecting with others online instead of in person.	Single-Item
15	I have negative thoughts about myself.	PED
16	Because of the heavy emotions I experience, I withdraw from others.	PED
17	I experience a sense of belonging and purpose through my religious beliefs. **(Reverse scored)**	PSD
18	I feel socially isolated.	PSD
**Instructions: Report the most accurate number on a scale of 0–5. 0** = **0, 1** = **1, 2** = **2, 3** = **3, 4** = **4, 5** = **5 or more**		
19	How many meaningful conversations do you have each day?	Single-Item
20	How many close relationships do you have?	Single-Item
21	How many hours a day do you normally spend on social media (e.g., TikTok, Facebook, Instagram, Snapchat, Tinder/dating sites)?	Single-Item
22	How many hours a day do you normally spend watching television, movies, or streaming content (e.g., Netflix, Disney+, Amazon Prime, YouTube)?	Single-Item
23	How many hours a day do you normally spend playing video games?	Single-Item
24	How many social events do you decline a week (for reasons other than studying, sleeping, self-care etc.) that result in you being alone?	Single-Item
25	How many religious services and/or activities do you meaningfully engage in per month?	Single-Item
**Instructions: Select the response that most accurately describes you or your situation. Scale consists of 1–10: 1** = **Not at all lonely;** **10** = **Extremely Lonely**		
26	How lonely do you feel right now?	Single-Item

Items 2, 5, 6, 13, and 17 are reverse scored. Category refers to if it loaded onto a factor (PED, psychoemotional disconnectedness; PSD, psychosocial disconnectedness) or if it should be used as a single-item. Contact the corresponding author for a convenient version of the instrument.

### 3.2 Participants

The total sample for this study consisted of 719 university students from a large mid-Atlantic religious university (*M*_age_ = 19.63, *SD*_age_ = 1.60). Inclusion criteria consisted of being ≥ 18 years old and a residential student at the university. The sample was predominantly female (75.3%). Regarding race/ethnicity, data was only collected for 627 out of the total 719 participants (missing from wave two). The race/ethnicity breakdown is as follows: 84.7% White, 4.1% Hispanic or Latinx, 3.8% Black, 3.5% Biracial or Multiracial, 3.3% Asian, 0.5% American Indian or Alaskan Native, and 0% Native Hawaiian or Pacific Islander. The overall sample displayed high homogeneity (mostly young adults, religious, female, and White).

### 3.3 Psychometric analysis

We used Exploratory Factor Analysis (EFA) and Principal Component Analysis (PCA) with Horn's (1965) Parallel-Analysis to establish the initial factors of the SCI. Both statistical techniques offer different explorative approaches, and the final model was selected based off of factors adhering closer to an existing theoretical framework. Structural Equation Modeling (SEM) was then conducted to further examine the factors of the SCI and to establish the model fit. The final model was evaluated using goodness-of-fit statistics, which included the adjusted goodness-of-fit index (AGFI), comparative fit index (CFI), Tucker-Lewis index (TLI), root mean square error of approximation (RMSEA), and standardized root mean square residual (SRMR) (Hu and Bentler, 1998). A Multi-Group Structural Equation Model (MG SEM) was then conducted to explore measurement invariance for the latent constructs across sex. The internal reliability was analyzed using Raykov's Rho (Composite Reliability) (Hair et al., 2019). Next, the average variance extracted (AVE) was calculated for each latent construct to examine the internal convergent validity (Fornell and Larcker, 1981), and heterotrait-monotrait ratio of correlations was calculated to examine the internal discriminant validity (Henseler et al., 2015). Subsequent correlations were calculated to examine the SCI's external convergent and divergent validity.

Data analyses were performed using SPSS v29.0.0.(241) (IBM Corp, 2023), STATA/BE (StataCorp, 2024), and Microsoft Excel.

### 3.4 Other measures

To examine the external convergent and divergent validity of the SCI, the following measures were used:

1. The UCLA LS three-item version (UCLA LS-3; Hughes et al., 2004) is a brief measure of loneliness based off of the revised UCLA LS (R-UCLA LS; Russell et al., 1980). A sample item is “how often do you feel that you lack companionship?” The three items are rated on a Likert-type scale ranging from one (hardly ever) to three (often), with higher scores indicating higher levels of loneliness. Hughes et al. (2004) reported good internal reliability (α = 0.72) and strong concurrent validity with the R-UCLA LS (*r* = 0.82) for the UCLA LS-3. In the current study, the UCLA LS-3 also demonstrated good internal reliability (α = 0.83). Data on this measure were collected from 292 participants (from wave three).
2. We included a single-item measure of loneliness right now (LRN) at the end of the SCI (item 26). The item was “How lonely do you feel right now?” The item was rated from one (not at all lonely) to ten (extremely lonely), with higher scores indicating higher levels of acute loneliness. LRN demonstrated strong concurrent validity with the R-UCLA LS (*r* = 0.77) and the UCLA LS-3 (*r* = 0.74). Data on this single-item measure were collected from all participants (from waves one, two, and three).
3. The Social Avoidance and Distress Scale (SADS; Watson and Friend, 1969) assesses anxiety and distress in the context of social situations. Participants respond to 28 items by answering if the item is true or false regarding their situation. A sample item is “I have no particular desire to avoid people.” After totaling the responses to items, higher scores indicate higher levels of social anxiety and distress. In the current study, the SADS demonstrated excellent internal reliability (α = 0.93). Data on this measure were collected from 91 participants (from wave two).
4. The Warwick-Edinburgh Mental Well-Being Scale (WEMWBS; Bass et al., 2016) measures one's subjective wellbeing. The WEMWBS is comprised of 14 items, which are rated on a Likert-type scale from one (none of the time) to five (all of the time). A sample item is “I've been feeling good about myself.” Higher scores on the scale indicate higher levels of wellbeing. In the current study, the WEMWBS demonstrated good internal reliability (α = 0.87). Data on this measure were collected from 91 participants (from wave two).
5. The Mindful Attention Awareness Scale (MAAS; Brown and Ryan, 2003) measures the present awareness and attention that one has in the current moment. Participants respond to 15 items on a Likert-type scale from one (almost always) to six (almost never). A sample item is “I could be experiencing some emotion and not be conscious of it until some time later.” Higher scores indicate higher levels of trait mindfulness. In the current study, the MAAS demonstrated good internal reliability (α = 0.88). Data on this measure were collected from 279 participants (from wave three).
6. The Healthy Lifestyle Screening for University Students (HLSUS; Dong et al., 2012) measures healthy lifestyle behaviors. The HLSUS consists of 38 items split into eight subscales, which include Exercise Behavior (α = 0.71), Regular Behavior (α = 0.72), Nutrition Behavior (α = 0.68), Health Risk Behavior (α = 0.43; omitted due to poor reliability), Health Responsibility (α = 0.55), Social Support (α = 0.54), Stress Management (α = 0.59), and Life Appreciation (α = 0.75). Items are rated on a Likert-type scale from one (never) to five (always). In the current study, the HLSUS total demonstrated good internal reliability (α = 0.81). Data on this measure were collected from 343 participants (from wave one).

## 4 Results

### 4.1 Data screening and assumptions

Analyses were conducted on all items to check data quality, descriptive statistics, and assumptions before further analysis. Of the initial data (*N* = 719), 652 responses (91.2%) had zero missing values. In total, there were 158 missing values (0.01%) across the 26 items on the SCI. Descriptive statistics were calculated for each item of the SCI (Supplementary Table 1). Items 1–18 (*M* = 3.09), 19–25 (*M* = 2.36), and 26 (*M* = 3.84) showed a slight bias toward lower scores in the current sample. The average inter-item correlation (*r*_s_) was 0.21, suggesting sufficient overlap between items while allowing for unique variance to be explained.

Data were then screened on assumptions regarding suitability for factor analysis on items 1–18. First, a Kaiser-Myer-Olkin was performed to provide a measure of sampling adequacy (MSA). The MSA = 0.90, which indicates that items were suitable for factor analysis (Kaiser, 1974). Next, Bartlett's test of sphericity was performed to test if the correlation matrix deviated from an identity matrix (i.e., all variables are unrelated). The test indicated that the correlation matrix was statistically significantly different from an identity matrix, χ2 (153) = 3,688, *p* < 0.001, which indicates that the variables are suitable for factor analysis (Bartlett, 1937).

### 4.2 Establishing the factors and model fit

To establish the initial factors of items 1–18 on the SCI, we performed an exploratory factor analysis (EFA) and a principal components analysis (PCA). The EFA was conducted using maximum likelihood extraction and an oblique rotation method (PROMAX), which assumes that the underlying factors are correlated. The number of factors retained was based on Horn's Parallel Analysis (Horn, 1965; O'Connor, 2000), which compares the mean eigenvalues and the eigenvalues at the 95th percentile created from 1,000 randomly generated datasets to those from the original dataset, as opposed to Kaiser's criteria (eigenvalues λ ≥ 1; Guttman, 1954; Kaiser, 1960). Overall, the EFA suggested five-factors which explained 42% of the variance, with six items (2, 5, 6, 13, 17, and 18) loading onto factor one, four items (4, 7, 8 and 12) loading onto factor two, two items (15 and 16) loading onto factor three, three items (7, 11, and 14) loading onto factor four, and one item (10) loading onto factor five. The PCA, which was also performed using an oblique rotation method (PROMAX) and with Horn's Parallel Analysis criteria, suggested two factors which explained 40% of the variance, with nine items (3, 4, 7, 8, 9, 10, 12, 15, and 16) loading onto factor one and eight items (1, 2, 5, 6, 11, 13, 17, and 18) loading onto factor two (Supplementary Table 2).

After reviewing the factors reported from each analysis, we decided that the two factors from the PCA had better scientific and theoretical support; therefore, subsequent analyses were performed using the two factors. Specifically, these two factors align closely with the theoretical framework established by Weiss's typology of loneliness, which describes two distinct dimensions of loneliness: emotional and social (Russell et al., 1984; Weiss, 1973). Perceived social disconnectedness, which is broader and more encompassing of aspects of social connection than loneliness, may also reflect these two (emotional and social) underlying components. Items 1 and 9 were removed prior to further testing from their respective factor for containing semantic content inconsistent with the rest of the items in addition to the lowest factor loadings (λ < 0.35).

**Figure 1:**
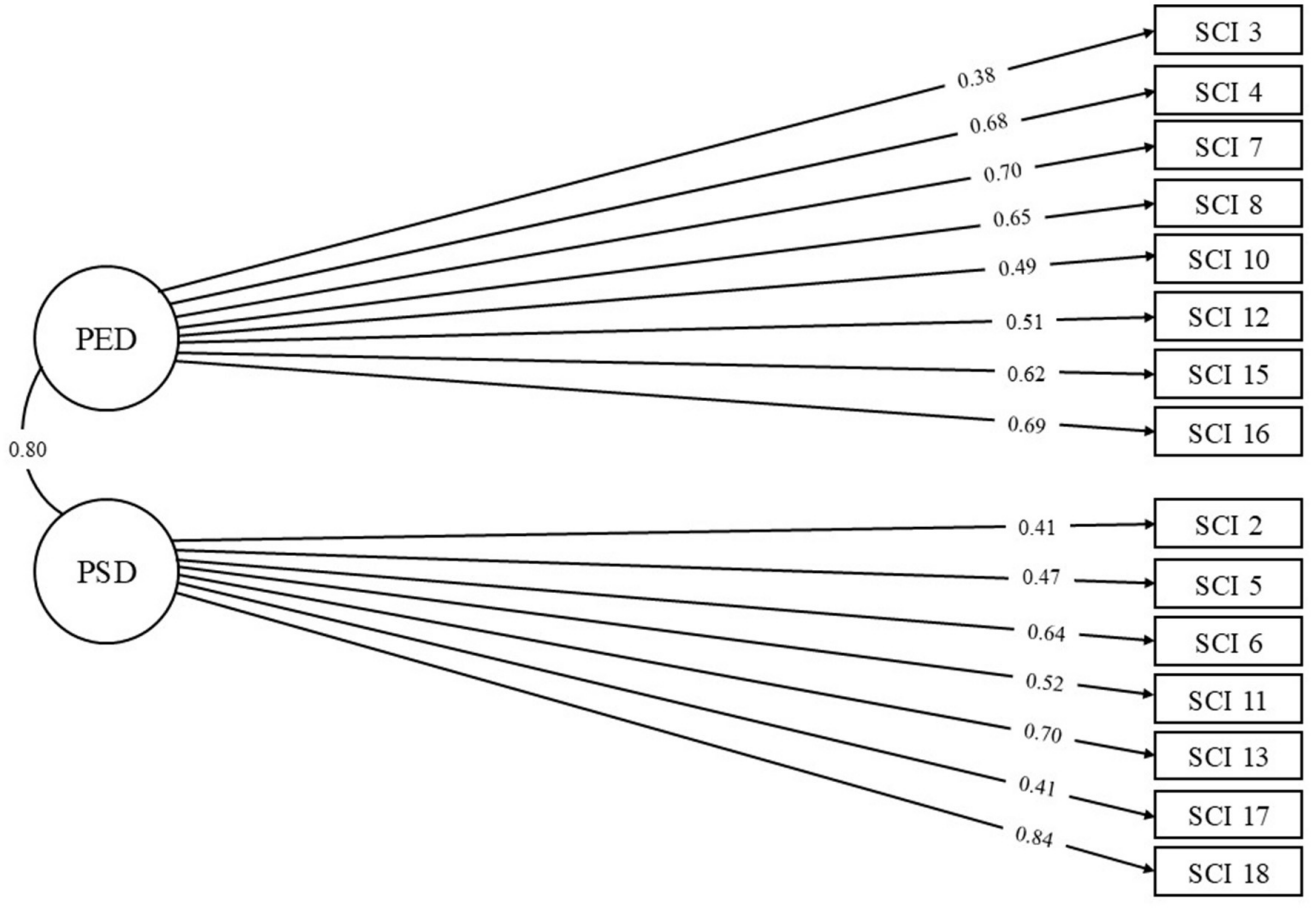
Final SEM model of the SCI. For clarity, error variances were excluded but are available upon request.

Next, structural equation modeling (SEM) was used in order to investigate the model fit. The final model consisted of eight items (3, 4, 7, 8, 10, 12, 15, and 16) on latent construct one and seven items (2, 5, 6, 11, 13, 17, and 18) on latent construct two. Overall, the model displayed satisfactory model fit indices [χ^2^ (82) = 315, *p* < 0.001; SRMR = 0.052; CFI = 0.985; TLI = 0.980; AGFI = 0.980] except for the RMSEA = 0.063 [95% CI (0.056, 0.071)], which was unsatisfactory (>0.050; Hu and Bentler, 1999). While the slightly elevated RMSEA can suggest model misspecification, given that the other fit indices are satisfactory, the overall model appears to perform well (Chen et al., 2008). The final model, which depicts the standardized factor loadings (ranging from 0.38 to 0.83; *M* = 0.58) between the indicators and the latent constructs, can be viewed in Figure 1.

We named the latent constructs based on the semantic content of each item that loaded onto it (Table 1). L1 (eight items) *Psychoemotional Disconnectedness* (PED) provides a measure of one's perception regarding their emotional experiences in the context of trying to connect, interact, or build relationships with others. All items on this construct are scored normally and higher scores reflect higher perceived social disconnectedness. L2 (seven items) *Psychosocial Disconnectedness* (PSD) provides a measure of one's perceived social skills, social opportunities, and social experiences. While most items (2, 5, 6, 13, and 17) are positively worded on this latent construct (e.g., I feel included by others), these items are reverse scored; thus, higher scores reflect higher perceived social disconnectedness. PED and PSD have a moderately strong relationship with one another (*r* = 0.58). Thus, while they are both capturing perceived social disconnectedness, they are measuring unique aspects of it.

### 4.3 Measurement invariance across sex

To establish the measurement invariance of the final model across sex (male *N* = 173, female *N* = 543), we performed a multi-group SEM. The results on measurement invariance found that no indicators showed sex bias (i.e., Wald test results across sex, *p* < 0.001). When the covariances and means of the latent variables were equal across groups, the multigroup SEM grouped by sex showed χ2 (217) = 638.12. The full model SEM resolved (scaled because of ordinal data) χ2(89) = 637. Therefore, the difference between the results across sex compared to the whole group equals: Δ χ2 (128) = 1.12, *ns*. Since Δ χ2 is viewed as one of the critical dimensions in evaluating group bias (Hair et al., 2019), results demonstrate that there is no statistically significant bias in the model due to sex differences. Both models (i.e., sex = male, sex = female) displayed high coefficients of determination (*CD* = 0.951); thus, each model accounted for 95.1% of the variation in the data.

### 4.4 Internal reliability and construct validity

The internal reliability of the constructs was calculated using Raykov's rho (Raykov and Shrout, 2002; Hair et al., 2022). PED (L1) displayed satisfactory reliability (ρ = 0.812) and PSD (L2) displayed satisfactory reliability (ρ = 0.776). Next, the internal convergent validity (i.e., whether the indicator variables converge to measure the same latent construct) was examined by calculating the average variance extracted (AVE) (Fornell and Larcker, 1981). PED (L1) and PSD (L2) did not meet the target cutoff of ≥0.50 (AVE = 0.358 and AVE = 0.345, respectively). However, given the high composite reliability and theoretical relevance for both of the latent constructs, we suggest that they each adequately converge to measure the underlying latent construct (PED and PSD) (Fornell and Larcker, 1981). For discriminant validity (i.e., whether the latent constructs can be differentiated from one another), the heterotrait-monotrait (HTMT) ratio of correlations was calculated (Henseler et al., 2015). The HTMT ratio of correlations (*r*_HTMT_ = 0.661) was below the cutoff of 0.85, which suggests that the latent constructs discriminate well from one another (i.e., measure unique aspects of perceived social disconnectedness). Additionally, in a small subset of participants from wave two (*N* = 43) who completed the SCI twice (seven days in between), both PED (*r* = 0.82) and PSD (*r* = 0.74) demonstrated acceptable test-retest reliability.

### 4.5 External convergent and divergent validity

Next, we examined the convergent and divergent validity of PED and PSD, which is how strongly the constructs relate to other similar (convergent) or dissimilar (divergent) constructs. As expected, both PED and PSD displayed moderate to strong relationships with LRN (*r*_PED_ = 0.56; *r*_PSD_ = 0.54) and the UCLA LS-3 (*r*_PED_ = 0.68; *r*_PSD_ = 0.70), which are brief measures of loneliness. Regarding other psychological variables, PED displayed moderate to strong relationships with SADS (*r* = 0.64), WEMWBS (*r* = −0.50), and MAAS (*r* = −0.46). PSD also displayed moderate to strong relationships with SADS (*r* = 0.52), WEMWBS (*r* = −0.64), and MAAS (*r* = −0.40). For self-reported health behaviors, the PED and PSD displayed weak to moderate relationships with the HLSUS total (*r*_PED_ = −0.29; *r*_PSD_ = −0.48). The PED and PSD displayed weak relationships (*r* < 0.40) with the HLSUS subscales, except for between PSD and Social Support (*r* = −0.45) and Life Appreciation (*r* = −0.48) (see Table 2). Thus, PED and PSD demonstrated high convergent validity with similar measures (e.g., UCLA LS-3, LRN, SADS, WEMWBS, MAAS) and high divergent validity with dissimilar measures (e.g., Exercise Behavior, Regular Behavior, Health Responsibility).

**Table 2:** Descriptive statistics and correlations between PSD, PED, and convergent/divergent measures.

**Measure**	***M***	***SD***	***N***	**α**	***r* (PED)**	***r* (PSD)**
PED	25.43	7.63	716	0.82	—	0.58^***^
PSD	20.91	5.80	714	0.78	0.58^***^	—
UCLA LS-3	5.03	1.81	279	0.83	0.68^***^	0.70^***^
LRN	3.84	2.29	694	—	0.56^***^	0.54^***^
SADS	10.30	7.73	91	0.93	0.64^***^	0.52^***^
WEMWBS	48.20	8.14	91	0.87	−0.50^***^	−0.64^***^
MAAS	3.63	0.79	279	0.88	−0.46^***^	−0.39^***^
HLSUS total	133.44	13.00	349	0.81	−0.29^***^	−0.48^***^
Exercise behavior	11.71	3.52	349	0.71	−0.11^*^	−0.18^**^
Regular behavior	12.39	3.23	349	0.72	−0.20^***^	−0.20^***^
Nutrition behavior	13.54	2.96	349	0.68	−0.10	−0.19^***^
Health responsibility	23.10	3.00	349	0.55	−0.01	−0.19^***^
Social support	22.75	2.96	349	0.54	−0.13^*^	−0.45^***^
Stress management	17.93	2.73	349	0.59	−0.28^***^	−0.35^***^
Life appreciation	18.34	2.90	349	0.75	−0.32^***^	−0.48^***^

* *p* < 0.05,

** *p* < 0.01,

*** *p* < 0.001.

PED, Psychoemotional Disconnectedness; PSD, Psychosocial Disconnectedness; UCLA LS-3, UCLA Loneliness Scale 3-item; LRN, Loneliness Right Now; SADS, Social Avoidance and Distress Scale; WEMWBS, Warwick-Edinburgh Mental Well-Being Scale; MAAS, Mindful Attention Awareness Scale; HLSUS, Healthy Lifestyle Screening for University Students. Exercise Behavior through Life Appreciation are subscales of the HLSUS.

Items 1, 9, and 14, which did not load onto the final model, and items 19–25, which were not used for the final model, can be used as single-item indicators of disconnectedness if someone scores high on PED, PSD, or alternative measures for social connectedness (e.g., loneliness, social support, etc.). All of these single-item measures, except for item 23, had statistically significant relationships with PED, PSD, UCLA LS-3, and LRN. Additionally, many of these items displayed statistically significant relationships with other psychological measures (SADS, WEMWBS, MAAS) and self-reported health behaviors (HLSUS) (see Table 3).

**Table 3:** Spearman correlations between single-item indicators and PSD, PED, and other measures.

	**Single-item numbers**									
**Measure**	**1**	**9**	**14**	**19**	**20**	**21**	**22**	**23**	**24**	**25**
PED	0.30^***^	0.26^***^	0.30^***^	−0.23^***^	−0.29^***^	0.12^**^	0.12^**^	0.06	0.24^***^	−0.14^***^
PSD	0.36^***^	0.22^***^	0.28^***^	−0.41^***^	−0.44^***^	0.12^**^	0.19^***^	0.11^**^	0.19^***^	−0.32^***^
UCLA LS-3	0.34^***^	0.28^***^	0.23^***^	−0.40^***^	−0.46^***^	0.18^**^	0.16^**^	0.11	0.27^***^	−0.15^*^
LRN	0.24^***^	0.21^***^	0.20^***^	−0.27^***^	−0.29^***^	0.18^***^	0.10^*^	0.10^*^	0.27^***^	−0.21^***^
SADS	0.21^*^	0.16	0.22^*^	−0.36^***^	−0.39^***^	0.08	0.13	0.04	0.31^**^	−0.21^*^
WEMWBS	−0.31^**^	−0.24^*^	−0.25^*^	0.32^**^	0.42^***^	−0.07	−0.22^*^	−0.06	−0.28^**^	0.23^*^
MAAS	−0.23^***^	−0.25^***^	−0.22^***^	0.26^***^	0.30^***^	−0.15^*^	−0.12^*^	−0.05	−0.21^***^	0.20^**^
HLSUS total	−0.17^***^	0.00	−0.22^***^	0.27^***^	0.23^***^	−0.16^**^	−0.24^***^	−0.13^*^	−0.13^*^	0.21^***^
Exercise behavior	−0.02	0.05	−0.09	0.05	0.14^**^	−0.03	−0.13^*^	−0.06	−0.06	0.07
Regular behavior	−0.05	−0.05	−0.16^**^	0.00	0.07	−0.17^**^	−0.24^***^	0.01	0.01	0.07
Nutrition behavior	−0.06	0.02	−0.19^***^	0.09	0.13^*^	−0.12^*^	−0.20^***^	−0.06	−0.06	0.07
Health responsibility	−0.06	0.05	−0.02	0.13^*^	0.06	0.05	0.07	−0.04	−0.04	0.08
Social support	−0.24^***^	0.04	−0.11^*^	0.29^***^	0.21^***^	0.06	−0.11^*^	−0.14^**^	−0.14^**^	0.27^***^
Stress management	−0.03	−0.08	−0.10	0.24^***^	0.14^*^	−0.17^**^	−0.15^**^	−0.08	−0.08	0.16^**^
Life appreciation	−0.24^***^	−0.01	−0.28^***^	0.29^***^	0.22^***^	−0.14^*^	−0.21^***^	−0.08	−0.08	0.19^***^

* *p* < 0.05,

** *p* < 0.01,

*** *p* < 0.001.

PED, Psychoemotional Disconnectedness; PSD, Psychosocial Disconnectedness; UCLA LS-3, UCLA Loneliness Scale 3-item; LRN, Loneliness Right Now; SADS, Social Avoidance and Distress Scale; WEMWBS, Warwick-Edinburgh Mental Well-Being Scale; MAAS, Mindful Attention Awareness Scale; HLSUS, Healthy Lifestyle Screening for University Students. Exercise Behavior through Life Appreciation are subscales of the HLSUS.

## 5 Discussion

### 5.1 Summary

While previous social connectedness measurement has focused solely on intensity, there is a need for a novel tool designed to capture not only intensity but also perceived contributors to social disconnectedness. To address this, the current study investigated the psychometric properties of the SCI. After preliminary descriptive and assumption analyses, an EFA and a PCA were performed to establish the initial factor-structure of items 1–18 on the SCI. Subsequent testing was performed using the two factor-structure established by the PCA. Next, SEM was used in order to examine the model fit and standardized factor loadings; the final model displayed satisfactory fit for all of the indices except for RMSEA, which was slightly unsatisfactory. After reviewing the content of each item on the respective latent construct, we named the first latent construct, Psychoemotional Disconnectedness (PED), and the second latent construct, Psychosocial Disconnectedness (PSD). The final model displayed measurement invariance across sex, satisfactory composite reliability, satisfactory discriminate validity, acceptable test-retest reliability, but both PED and PSD displayed low convergent validity (AVE < 0.5). Because PED and PSD showed satisfactory composite reliability, we propose that these constructs have adequate convergent validity (Fornell and Larcker, 1981) and therefore measure the same overall construct. PED and PSD displayed high external convergent and divergent validity, with higher correlations with measures of similar constructs and lower correlations with measures of dissimilar constructs. Additionally, the single-item indicators, which were not a part of PED or PSD, also demonstrated high external convergent and divergent validity.

### 5.2 Interpreting the theoretical model of the SCI: from perceived social disconnectedness to connectedness

**Figure 2:**
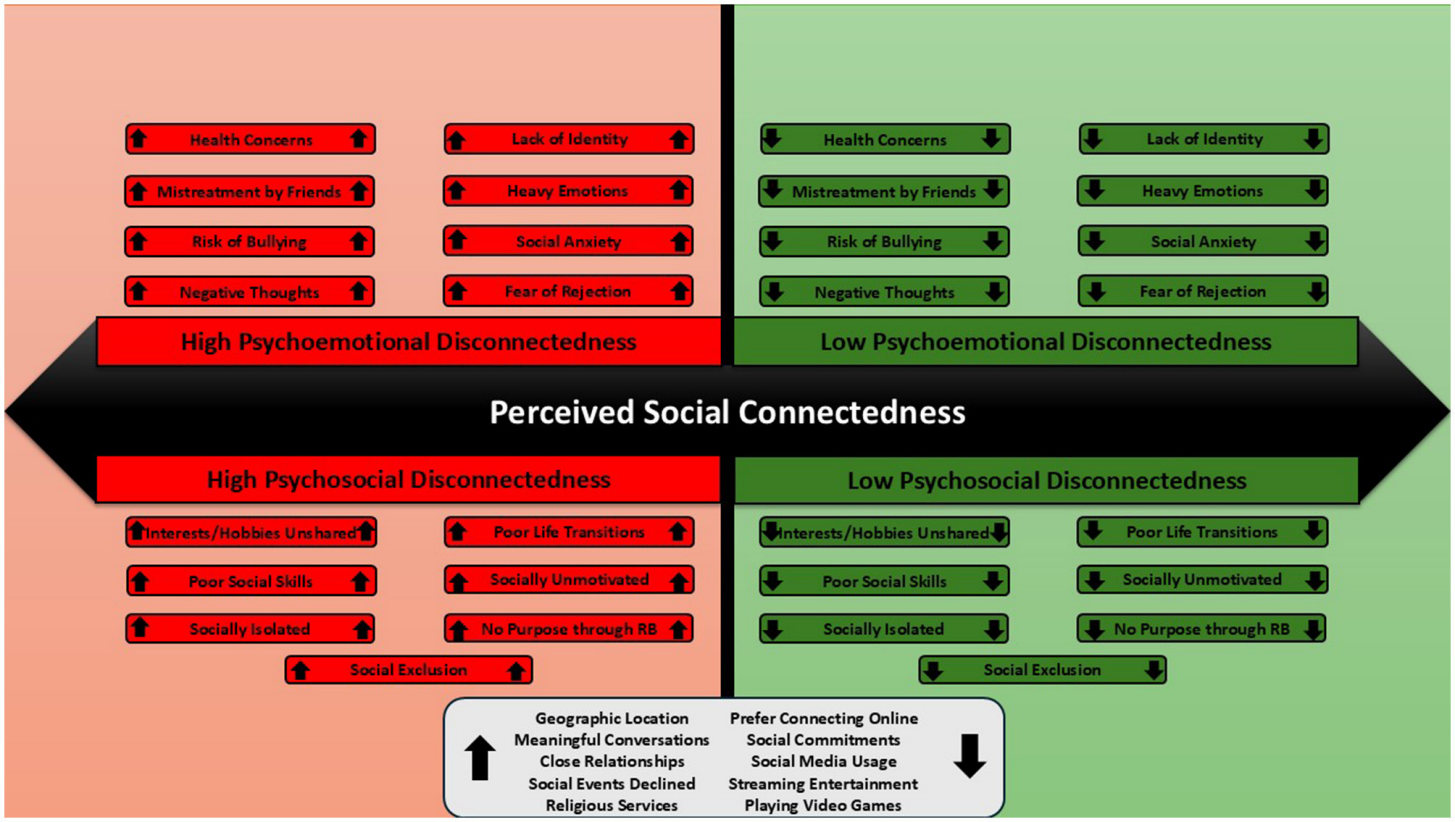
Final theoretical model of the SCI. The arrow direction indicates high scores (pointing up) or low scores (pointing down).

The overall model of the SCI consists of two correlated latent constructs, PED and PSD, with additional single-item indicators of (dis)connectedness that may further detail an individual's social experience. While the two latent constructs are framed negatively (i.e., disconnectedness), the instrument name, the Social *Connectedness* Instrument, is framed positively (i.e., connectedness). Similar to the mental health continuum, which ranges from languishing (negative) to flourishing (positive) (Keyes, 2002), the theoretical continuum of social connectedness ranges from disconnectedness (negative) to connectedness (positive) (Holt-Lunstad, 2024). The SCI focuses on a continuum of *perceived* social connectedness, which ranges from low perceived connectedness (i.e., high disconnectedness) to high perceived connectedness (i.e., low disconnectedness). Thus, higher scores on PED and PSD indicate higher perceived disconnectedness (lower connectedness), while lower scores indicate lower perceived disconnectedness (higher connectedness) (see Figure 2).

#### 5.2.1 Psychoemotional disconnectedness and psychosocial disconnectedness

PED and PSD form related but unique constructs of perceived social disconnectedness. While these are similar constructs to emotional and social loneliness, as established by Weiss (1973) and measured using the DJGLS (de Jong-Gierveld and Kamphuis, 1985) and SELSA (DiTommaso and Spinner, 1993), they are distinct in that they measure the broader construct of *perceived social disconnectedness* and provide valuable insight into modifiable contributors to the experience of feeling disconnected from others (e.g., loneliness, social support, social cohesion). PED is made up of items that assess health concerns (item 3) (Carter et al., 2015; Tuncay et al., 2018), fear of rejection (item 4) (Zlomke et al., 2016), social anxiety (item 7) (Danneel et al., 2019; O'Day et al., 2021), bullying (item 8) (Bayat et al., 2021), mistreatment by friends (item 10) (Jones et al., 2020), lack of identity (item 12) (Kaniušonyte et al., 2019), negative thoughts (item 15) (Besser et al., 2022), heavy emotions (item 16) (Buecker et al., 2021; Gum et al., 2017; Lim et al., 2016) in the context of how lonely or socially disconnected it makes the person feel (see Table 1). The items converge together to provide insight into the overall psychoemotional aspects of perceived social disconnectedness, while still providing insight into potentially modifiable psychoemotional contributors at an item-by-item level. Psychosocial Disconnectedness (PSD) is made up of items that assess life transitions (item 2) (Laursen and Hartl, 2013; Sundqvist et al., 2024), interests/desires/hobbies (item 5) (Mak et al., 2023; Martin et al., 2013), social skills/confidence (item 6) (Floyd and Woo, 2020; Lodder et al., 2016), social motivation (item 11) (Nikitin and Freund, 2017), feeling included (item 13) (Park and Baumeister, 2015), experiencing belonging/purpose through religious beliefs (item 17) (Nezlek, 2021), and perceived isolation (item 18) (Cacioppo et al., 2010) in the context of how socially disengaged and disconnected they make the person feel (items 2, 5, 6, 13, and 17 are reverse-scored; see Table 1). The items converge together to provide insight into the overall psychosocial aspects of perceived social disconnectedness, while still providing insight into potentially modifiable psychosocial contributors at an item-by-item level. PED and PSD demonstrated high external convergent validity, displaying strong relationships with the UCLA LS-3 (Hughes et al., 2004) and item 26 on the SCI. Additionally, both PSD and PED displayed moderate to strong relationships with social anxiety, wellbeing, and mindfulness.

PED and PSD have a moderate positive association with one another, which suggests that as someone displays higher PED, they also tend to display higher PSD. This finding lines up with previous social connectedness instruments which also have associated factors, such as between social and emotional loneliness on the DJGLS (de Jong-Gierveld and Kamphuis, 1985) and SELSA (DiTommaso and Spinner, 1993). However, it is possible for someone to display a high score for PED and a low score for PSD since these are related but unique constructs. Thus, someone can theoretically have feelings of disconnectedness and connectedness simultaneously. For example, someone could experience emotional disruptions (e.g., social anxiety, fear of rejection) that make them feel disconnected from others (high PED) while having sufficient social opportunities (e.g., shared interests/hobbies, belonging through religious beliefs) that make them feel connected to others (low PSD). However, given the relationship between PED and PSD, it is more likely that one will feel disconnected/connected both emotionally and socially.

#### 5.2.2 Other single-item indicators of disconnectedness

Of the 26 items on the SCI, three items did not load onto a factor (1, 9, and 14) and eight items were not included in the factor analysis (items 19–26). Although these items were not included in the final factors of the SCI, we recommend including them still if there are no time-constraints as they provide more information about an individual's experience with social connectedness. Item 1 examines how geographic location may impact a person's ability to connect with others. While objective social isolation and loneliness typically have a weak relationship with one another (Lennartsson et al., 2022), people may perceive that their specific geographical area offers limited opportunities to connect with others, which can contribute to feelings of disconnectedness (Victor and Pikhartova, 2020). Item 9 examines how commitments may impede one's ability to have a social life. As people develop across the lifespan, it is essential to consider how specific commitments (e.g., job, school, family) may reduce the ability or time available for building meaningful social connections (McKenna-Plumley et al., 2023). Question 19 (meaningful conversations), question 20 (close relationships), and question 25 (meaningful religious services attended) provide brief measures focused on more quantifiable components of social connection. Higher levels for these constructs are associated with better overall social connectedness (Block et al., 2022; Hastings, 2016; Okabe-Miyamoto et al., 2024). Conversely, question 24 (social events declined) provides a brief quantifiable measure of how isolated one is choosing to be by turning down invitations for connection, which can be related to higher feelings of perceived isolation (Santini et al., 2020). Item 14 (preference for connecting online), question 21 (social media usage), question 22 (streaming entertainment), and question 23 (playing video games) all provide information about someone's technology use. While technology use is increasingly prevalent and not always an indicator of poor social connectedness, studies have demonstrated a relationship between fewer in-person interactions and disconnectedness (Twenge et al., 2019), decreasing social media usage and improvements in feelings of loneliness (Hunt et al., 2018), higher binge-watching behavior and higher loneliness (Gabbiadini et al., 2021), and online gaming addiction and higher loneliness (Gao et al., 2024). Each of these single-item indicators of disconnectedness had statistically significant relationships with the UCLA LS-3 (Hughes et al., 2004) (except item 23), LRN (item 26), PED (except item 23), and PSD, suggesting that they provide valuable insight into a person's experience with social disconnectedness. However, due to their single-item nature (i.e., no measure of reliability), they must be interpreted with caution and may need longer-form follow-up assessments (e.g., interviews, longer self-report instruments) if individuals score high.

#### 5.2.3 Advantages

Previous research suggests that intervention plans tailored to a person's specific experience may be more effective at increasing social connectedness and reducing feelings of disconnectedness (Eccles and Qualter, 2021; Hickin et al., 2021; Masi et al., 2011). However, the most widely used instrument to measure loneliness, the UCLA LS Version 3 (Russell, 1996), provides a unidimensional measure of loneliness that captures how lonely someone is without providing many details about possible reasons for their experience. The SCI provides a novel theoretical framework in which social connectedness interventions can better be tailored to a person's specific experience of feeling disconnected. Instead of a general intervention, such as a social skills or mindfulness training, being assigned arbitrarily to individuals with poor connectedness, professionals (e.g., practitioners, researchers, educators, etc.) can now measure and intervene on two correlated but distinct latent constructs of perceived social disconnectedness. It is recommended to first target the latent construct (PED or PSD) with a higher score or to combine approaches if scores are equal (e.g., a socially and emotionally focused intervention). For example, if someone scores higher on PSD, one should further examine the scores of each item that form the latent construct and develop a socially focused intervention that will help the person to overcome their specific challenges. Perhaps the individual scored highest on item 5 (interests/desires/hobbies), item 6 (social skills/confidence) and item 13 (feeling excluded). In this case, an intervention for this individual may first try to increase feelings of social connectedness by helping the person to improve their social skills and social confidence in relationships, while offering direction for finding and connecting with other people who share similar interests/hobbies. The additional single-item indicators of (dis)connectedness may also offer direction for personalized interventions that are more quantifiable/behavioral (e.g., reducing technology use, increasing meaningful conversations). However, since this is a novel theoretical framework for improving perceived social connectedness, data needs to be collected to examine the effectiveness of using an individualized approach informed by the SCI as opposed to the typical population-based intervention approach.

### 5.3 Limitations and future directions

The study's most significant limitation is the homogeneous sample, which included a sample of college students who were mainly religious (Christian), White, and female. Due to the sampling limitation, the SCI has limited external validity across other demographics. This could have impacted the results, such as by influencing which specific items loaded onto each factor. For example, item 17 (purpose through religion) loaded onto PSD, but may not load onto the factor in other non-religious samples. Due to the sampling limitation, the SCI has limited external validity across other demographics. Thus, a future study should investigate the external and cross-cultural validity with a more diverse sample, including people of different ages, socioeconomic statuses, races/ethnicities, countries, and health statuses (clinical populations). Next, only a couple of secondary measures were included to capture external convergent/divergent validity. A subsequent study should examine how the SCI is related to other established measures of social connectedness (loneliness, social support, and social isolation) and mental health (depression, anxiety, stress, self-esteem, etc.). Lastly, there is no data to support that these latent constructs (PED and PSD) and other single-item indicators help to provide a more personalized intervention for reducing loneliness and promoting social connectedness. Therefore, a future longitudinal study should examine if tailoring an intervention based off of the SCI is better at improving social connectedness as compared to assigning someone to any social connectedness intervention at random.

## 6 Conclusion

In efforts to reduce loneliness and promote social connection, it is important to investigate novel instruments that provide further depth. Overall, the testing on the SCI revealed two latent constructs (PED and PSD) in addition to other single-item indicators of disconnectedness, which were well-supported statistically and theoretically. However, in order to generalize this model to different demographics, further studies need to investigate how the SCI performs amongst different samples. While there are established measures of loneliness and social connectedness, the SCI provides further insight into one's experience with perceived social disconnectedness that may be useful for implementing personalized social connectedness instruments.

## Data Availability

The raw data supporting the conclusions of this article will be made available by the authors, without undue reservation.
