# Supplementary material for: Psychometric analysis of the social connectedness instrument
Source: Front Psychol. 2025 Jul 11;16:1565267. doi: 10.3389/fpsyg.2025.1565267 (PMC12290410; doi:10.3389/fpsyg.2025.1565267)
Supplement: Supplementary file 1 [file Table_1.docx]

**Table 1**

*Descriptive Statistics*

| # | Item | *N* | *M* | *SD* | *Med* | *Min* | *Max* | *Sk* | *Ku* |
| --- | --- | --- | --- | --- | --- | --- | --- | --- | --- |
| 1 | Where I live limits my ability to have consistent positive social interactions. | 719 | 2.79 | 1.47 | 3.00 | 1.00 | 7.00 | 0.70 | -0.07 |
| 2 | Recent transitions have made me feel more socially connected. **(Reverse scored)** | 717 | 3.49 | 1.29 | 3.00 | 1.00 | 7.00 | 0.38 | 0.18 |
| 3 | Health concerns make it difficult for me to connect with others. | 718 | 2.10 | 1.42 | 2.00 | 1.00 | 7.00 | 1.32 | 1.13 |
| 4 | I am concerned people will reject me. | 719 | 4.06 | 1.68 | 4.00 | 1.00 | 7.00 | -0.10 | -0.81 |
| 5 | My interests, desires, or hobbies are shared by the people around me. **(Reverse scored)** | 719 | 3.10 | 1.07 | 3.00 | 1.00 | 7.00 | 0.56 | 0.43 |
| 6 | I feel confident in my ability to initiate relationships with others. **(Reverse Scored)** | 718 | 3.20 | 1.33 | 3.00 | 1.00 | 7.00 | 0.31 | -0.22 |
| 7 | I feel anxious in social situations. | 719 | 4.06 | 1.49 | 4.00 | 1.00 | 7.00 | 0.13 | -0.50 |
| 8 | The risk of being bullied outweighs my desire to build friendships. | 719 | 2.18 | 1.30 | 2.00 | 1.00 | 7.00 | 1.07 | 0.72 |
| 9 | My commitments (e.g., work, school, extracurriculars) interfere with my social life. | 719 | 4.23 | 1.39 | 4.00 | 1.00 | 7.00 | -0.01 | -0.21 |
| 10 | I think I am treated poorly by my friends. | 719 | 2.36 | 1.17 | 2.00 | 1.00 | 7.00 | 0.72 | 0.27 |
| 11 | I lack the motivation to invest in deeper relationships. | 719 | 2.78 | 1.39 | 3.00 | 1.00 | 7.00 | 0.53 | -0.33 |
| 12 | To fit in, I change how I act around people. | 719 | 3.05 | 1.45 | 3.00 | 1.00 | 7.00 | 0.31 | -0.45 |
| 13 | I feel included by others. **(Reverse scored)** | 718 | 3.08 | 1.09 | 3.00 | 1.00 | 7.00 | 0.58 | 0.79 |
| 14 | I prefer connecting with others online instead of in person. | 718 | 2.22 | 1.34 | 2.00 | 1.00 | 7.00 | 1.14 | 0.99 |
| 15 | I have negative thoughts about myself. | 719 | 4.08 | 1.50 | 4.00 | 1.00 | 7.00 | -0.06 | -0.49 |
| 16 | Because of the heavy emotions I experience, I withdraw from others. | 717 | 3.50 | 1.51 | 4.00 | 1.00 | 7.00 | 0.12 | -0.64 |
| 17 | I experience a sense of belonging and purpose through my religious beliefs. **(Reverse scored)** | 719 | 2.32 | 1.29 | 2.00 | 1.00 | 7.00 | 1.13 | 1.34 |
| 18 | I feel socially isolated. | 718 | 3.00 | 1.37 | 3.00 | 1.00 | 7.00 | 0.37 | -0.39 |
| 19 | How many meaningful conversations do you have each day? | 711 | 2.94 | 1.27 | 3.00 | 0.00 | 5.00 | 0.15 | -0.71 |
| 20 | How many close relationships do you have? | 712 | 4.12 | 1.14 | 5.00 | 0.00 | 5.00 | -1.00 | -0.11 |
| 21 | How many hours a day do you normally spend on social media (e.g., TikTok, Facebook, Instagram, Snapchat, Tinder/dating sites)? | 691 | 2.38 | 1.40 | 2.00 | 0.00 | 5.00 | 0.23 | -0.74 |
| 22 | How many hours a day do you normally spend watching television, movies, or streaming content (e.g., Netflix, Disney+, Amazon Prime, YouTube)? | 713 | 1.55 | 1.31 | 1.00 | 0.00 | 5.00 | 0.92 | 0.44 |
| 23 | How many hours a day do you normally spend playing video games? | 693 | 0.38 | 0.85 | 0.00 | 0.00 | 5.00 | 2.74 | 8.26 |
| 24 | How many social events do you decline a week (for reasons other than studying, sleeping, self-care etc.) that result in you being alone? | 693 | 1.13 | 1.18 | 1.00 | 0.00 | 5.00 | 1.06 | 0.82 |
| 25 | How many religious services and/or activities do you meaningfully engage in per month? | 696 | 4.05 | 1.44 | 5.00 | 0.00 | 5.00 | -1.43 | 0.94 |
| 26 | How lonely do you feel right now? | 694 | 3.84 | 2.29 | 3.00 | 1.00 | 10.00 | 0.55 | -0.68 |

*Note*. *N* – sample size; *M* – sample mean; *SD* – sample standard deviation; *Med* – median; *Min* – minimum score; *Max* – maximum score; *Sk* – skewness; *Ku* – kurtosis.

| **Table 2**  *Principal Component Analysis Factor Loadings* | | | |
| --- | --- | --- | --- |
|  | Factor | |  |
| Item | 1 | 2 | Uniqueness |
| 1 |  | 0.301 | 0.832 |
| 2 |  | 0.780 | 0.554 |
| 3 | 0.536 |  | 0.761 |
| 4 | 0.824 |  | 0.417 |
| 5 |  | 0.720 | 0.568 |
| 6 |  | 0.617 | 0.543 |
| 7 | 0.608 |  | 0.515 |
| 8 | 0.650 |  | 0.550 |
| 9 | 0.345 |  | 0.879 |
| 10 | 0.351 |  | 0.739 |
| 11 |  | 0.468 | 0.667 |
| 12 | 0.618 |  | 0.641 |
| 13 |  | 0.696 | 0.430 |
| 14 |  |  | 0.819 |
| 15 | 0.812 |  | 0.441 |
| 16 | 0.726 |  | 0.421 |
| 17 |  | 0.642 | 0.657 |
| 18 | 0.398 | 0.501 | 0.372 |

*Note*. Factor loadings > 0.30 were initially retained.
